# Availability, Affordability, Access, and Pricing of Anti-cancer Medicines in Low- and Middle-Income Countries: A Systematic Review of Literature

**DOI:** 10.3389/fpubh.2021.628744

**Published:** 2021-04-30

**Authors:** Phyllis Ocran Mattila, Rabbiya Ahmad, Syed Shahzad Hasan, Zaheer-Ud-Din Babar

**Affiliations:** ^1^Department of Pharmacy, University of Huddersfield, Huddersfield, United Kingdom; ^2^Faculty of Pharmacy, The Islamia University of Bahawalpur, Bahawalpur, Pakistan

**Keywords:** pricing, availability, affordability, access, anti-cancer medicines, low-income and middle-income countries

## Abstract

**Background:** Cancer is the second leading cause of death globally accounting for more than half of deaths in Low- and Middle-Income Countries (LMICs). Cancer treatment is expensive and the high prices of cancer medicines have a huge impact on access in LMICs. Scarcity of pricing or affordability data is one of the major barriers in the development of effective and transparent pricing policies in LMICs. This study aimed to conduct a systematic review of the literature regarding pricing, availability, affordability, and access to anti-cancer medicines in LMICs.

**Method:** A systematic search was conducted across six electronic databases: PubMed, Medline/CINAHL (EBSCO), Web of Science, Springer Links, Scopus, and Google Scholar. The literature (from 2015 to 2020) was reviewed to identify original research articles published in English.

**Results:** A total of 13 studies were included in the review with some having multiple outcomes: five studies on pricing, four studies addressed affordability, five studies reported on availability, and four studies on access to anti-cancer medicines. The studies showed that in LMICs, there are wide variations in cancer prices and availability amongst the medicine brands and across different countries, with less affordability by patients with low-income levels, sometimes leading to treatment abandonment.

**Conclusion:** Given the importance of medicine availability and prices in patient access and medicine buying capacity of governments, multi-pronged policy and program approaches by multiple stakeholders are needed to ensure access to cancer medicines.

## Introduction

The global cancer burden is estimated to have risen to 18.1 million new cases and is responsible for an estimated 9.6 million deaths in 2018 (1). Globally, about one in six deaths is due to cancer (1). Unless a greater effort is done to alter the course of the disease, this number is expected to rise to close to 30 million new cases by 2040 (2). About 70% of deaths from cancer occur in Low- and Middle-Income Countries (LMICs) (1). Despite having almost 80% of the burden as measured by disability-adjusted life-years (DALYs), LMICs have less than an estimated 5% share of the global resources for combating cancer (3).

There are concerns about the lack of adequate access to both new and off-patent essential cancer medicines, with soaring prices cited as a main contributory factor impacting affordability for the large populations in LMICs (2–4). For example, a course of standard treatment (doxorubicin, cyclophosphamide, docetaxel, trastuzumab) for early-stage human epidermal growth factor receptor 2 positive (HER2+) breast cancer would cost about 10 years of average annual wages in India and South Africa (2).

A World Health Organization (WHO) technical report showed that countries with a lower national income had lower availability of anti-cancer medicines, or availability only with higher out-of-pocket patient payments, especially for higher-cost medicines, including targeted therapies (3). It was reported that 32.0 and 57.7% of cancer medicines on the essential medicine list were available in lower-middle-income and low-income countries, respectively, only if patients were willing to incur their full costs (3).

The WHO's Model of Essential Medicines List (EML) for adults and Essential Medicines List for children (EMLc) presents a list of minimum medicine needs for a basic health-care system, listing the most efficacious, safe, and cost-effective medicines for priority conditions. It may serve as a guide to help countries in the development of the national and institutional essential lists and reimbursable lists for the public sector to improve the accessibility, availability, and affordability of essential medicines needed to treat curable adult and childhood cancers respectively (5).

In LMICs, large proportions of the population have limited access to medicines, either because of poor availability or because patients must pay for their prescriptions. In the absence of government reimbursements, insurance, or any exclusive access schemes in LMICs, many patients must bear the cost of the treatment. This in turn forces them into deprivation, poverty, or early death.

Access to medicines for patients in LMICs is constrained by government underfunding of medicines and institutional weaknesses in the pharmaceutical sector for procuring and supplying medicines that contribute to poor inventory control and potentially suboptimal utilization of these products (3).

Current pricing policies (or the lack thereof) have led to considerable variability in the prices of cancer medicines within a country and across regions (3). Scarcity of pricing or affordability data is one of the major barriers in the development of effective and transparent pricing policies in LMICs. For fair and transparent pricing of cancer medicines, systems should be put in place to generate reliable and quality data to guide the choice of the most suitable pricing model for cancer medicines.

Prior to designing effective interventions that promote access to anti-cancer treatment, it is necessary to understand the factors affecting access, pricing, affordability, availability of cancer medications. Although research on access, pricing, affordability, availability of anti-cancer medications has been reported in some LMICs, such information has not been collated and synthesized to show the overall landscape. To the best of our knowledge, there is no systematic review(s) on the availability, affordability, and pricing of anti-cancer medicines in LMICs. In this review, a systematic literature review was conducted aiming to provide an overview of access, pricing, affordability, availability of anti-cancer medicines in current literature in the LMICs context.

## Materials and Methods

This systematic review was registered with the International Prospective Register of Systematic Reviews, PROSPERO (6), and was assigned the following registration number: CRD42020214365.

### Search Strategy

The Preferred Reporting Items for Systematic Reviews and Meta-Analyses (PRISMA) guidelines for conducting systematic reviews were followed (7). The search was conducted in May 2020 in six databases namely: Medline/CINAHL EBSCO, PUBMED, Web of Science, Google Scholar, Springer link, and Scopus to identify published peer-reviewed articles in English. The papers published between January 2015 to May 2020 were included in this review. The search key terms were availability, affordability, prices, pricing, cancer medicines, cancer medication, anti-cancer medicines, oncology medicines, low-income countries, developing countries, middle-income countries, LMICS, access, and accessibility. We have used various combinations of the above search terms.

References of retrieved articles were assessed for relevant articles that our searches may have missed.

### Inclusion/Exclusion Criteria

Studies reporting on availability, affordability, access, and pricing were eligible for inclusion (i.e., what the study had to fulfill in order to be included in the systematic review), according to the following definitions as reported in the literature. Affordability: the ability to purchase a necessary quantity of a product or level of service without suffering undue financial hardship. Affordability was also considered in terms of the value of the product, within the context of healthcare system budgets and whether products are affordable in a given country based on economic factors (3, 8–11). Availability: A patient can obtain when needed, for free or for a fixed fee, a pharmaceutical product that is listed on the national formulary (3, 10). Price: Price components, observed or derived, along the value chain from the manufacturer, distributor, service providers to patients. Pricing also refers to the price paid by the government, wholesalers, retailers, other purchasers, and consumers to procure medicines (3, 10). Access/Accessibility: is the ability of an individual to access care when needed (12). Low- and middle-income Countries: For the current 2021 fiscal year, low-income economies are defined as those with a Gross National Income (GNI) per capita, calculated using the World Bank (WB) Atlas method, of $1,035 or less in 2019; lower-middle-income economies are those with a GNI per capita between $1,036 and $4,045; upper-middle-income economies are those with a GNI per capita between $4,046 and $12,535 (13).

Inclusion criteria were: (a) studies on the availability of anti-cancer medicines; (b) studies on the affordability of anti-cancer medicines; (c) studies on the pricing of anti-cancer medicines; (d) studies on the access of anti-cancer medicines; (e) studies conducted in LMICs (13); (f) studies published as original research articles; (g) studies published between January 2015 to May 2020; (h) studies published in English; (i) studies with the available full text. The search was limited to original research articles in peer-reviewed journals.

Exclusion criteria: Magazines, reviews, editorial letters, lectures, and other publications that did not provide the relevant data or any of the outcomes listed as part of the inclusion criteria were excluded, as well as those articles not available as full text.

### Quality Assessment

To avoid bias in the study, a strict selection of the articles was made following approved guidelines (14) and pre-defined inclusion criteria to provide reliable data. The Newcastle-Ottawa Scale (NOS) for assessing the quality of non-randomized studies was used to assess the quality of included studies (15). The title and abstract of all retrieved articles were reviewed by the lead author (PO) for relevance and internal validity. Subsets of research results were checked independently by a second author (ZB or RH) for inclusion and exclusion. The final inclusion of studies was based on consensus among the review team and was listed along with important characteristics and results of each study. If there was any ambiguity or conflict with regards to the paper, it was resolved by the review team through discussion, and the consensus was developed. We do not plan to undertake a meta-analysis, but have a narrative summary describing the included studies main findings and outcome measures.

### Screening and Data Extraction

The initial results were collated onto a spreadsheet and abstracts screened for eligible studies. Abstracts from all selected articles in the first stage were read to determine their relevance. Duplicate articles were removed. The results were then peer-reviewed for errors in spelling, syntax, and line combinations. All articles considered potentially eligible were read in full to determine their relevance according to the inclusion criteria and if the study focused on affordability, availability, pricing, and access to anti-cancer medicines in LMICs.

At the full-text review stage, studies not meeting the inclusion criteria were excluded. Information extracted on the studies included details on the title, author, year of publication, data collection period, sample size, study details, methodology/assessment, key points, outcome measures, and the main findings of the study. The eligible full-text articles were finalized after discussion with the review team and filled into the data extraction sheet. All included studies were listed in the review, along with descriptions of their key characteristics.

### Analysis

We reviewed the literature systematically to ensure that a narrative synthesis produced was sourced from the most complete collection of relevant literature possible. Thematic analysis of the articles was conducted, and relevant sub-categories were created for examination until no more themes were identified and saturation was deemed to be reached.

## Results

**Figure 1:**
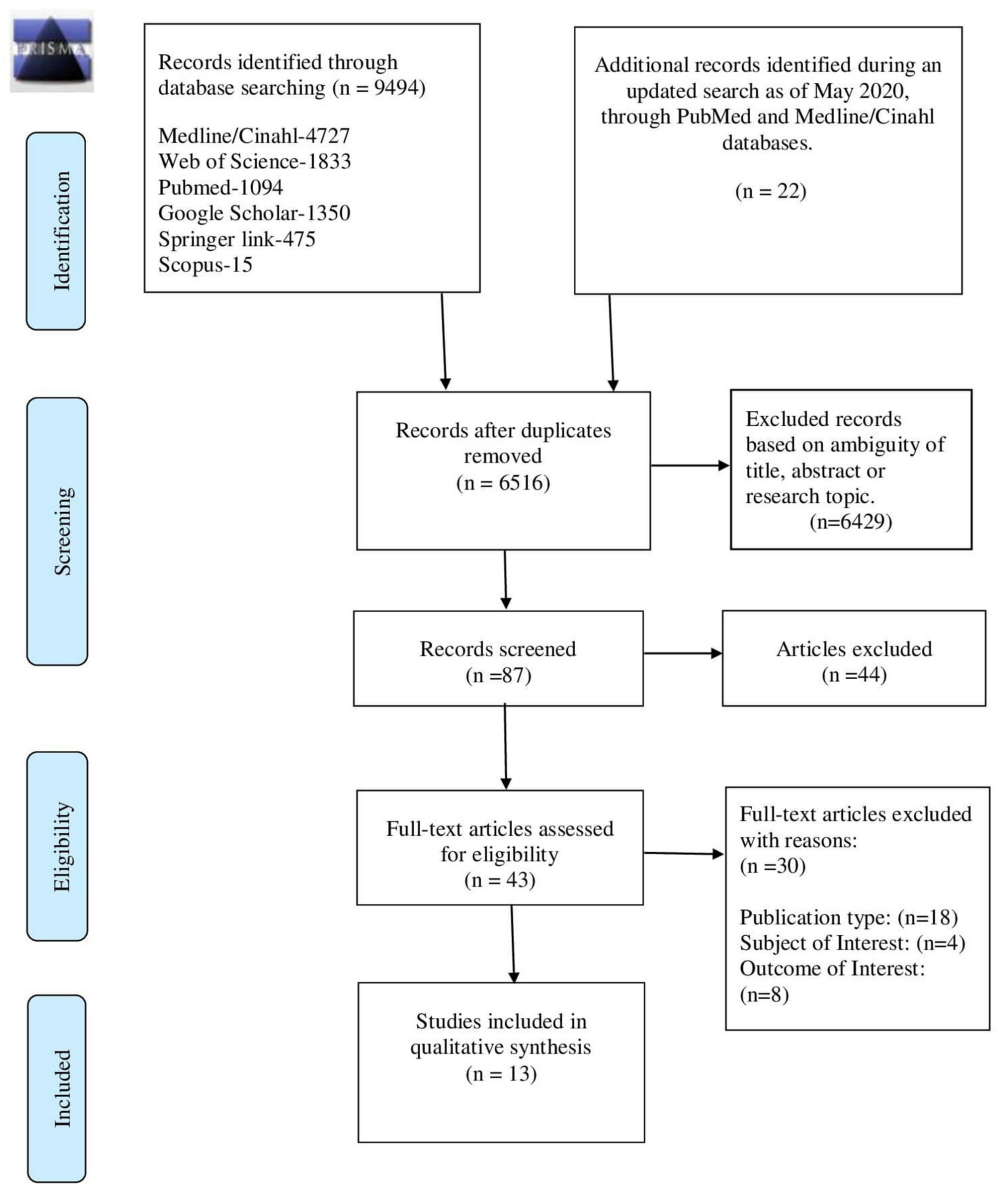
Study selection flow chart—PRISMA 2009 flow diagram (7).

The search of six electronic databases in early May 2020 yielded a total of 9,516 articles comprising 9,494 abstracts, and an additional 22 abstracts from an updated search as of the end of May 2020, with the removal of 3,000 duplicate abstracts, and 6,429 excluded based on ambiguity of title, abstract, or research topics (Figure 1). In total, 87 articles were identified as being potentially relevant to the review objectives, and full-text versions were obtained. Of the 87 potentially relevant articles, 44 were excluded and 43 full-text articles were assessed in-depth for eligibility based on the defined criteria and following the Cochrane guidelines (14). After application of inclusion and exclusion criteria, 13 studies were finally retained for qualitative synthesis by the review team.

Overall, 13 studies were included in the review with some having multiple outcomes: five studies (16–20) were on the pricing of anticancer medicines, four studies (4, 18, 21, 22) addressed affordability of anti-cancer medicines, and five studies reported (8, 18, 20, 22, 23) on the availability of anticancer medicines and overall four studies (8, 24–26) was on access to anti-cancer medicines.

The relationship between the included studies, overview of the methodology, main findings and outcome categories generated by this analysis on pricing, availability, affordability, and access to anti-cancer medicines are described in Table 1.

**Table 1:** Study details/findings.

**Reference**	**Treatment**\**population**\** sample size**	**Study details**	**Method**\**assessment details (assessment of accessibility, availability, costs)**	**Outcomes/variables measured**	**Main findings of the study**
(4)	Medicines-8 patented cancer drugs: bevacizumab, bortezomib, dasatanib, erlotinib, imatinib, pemetrexed, rituximab, and trastuzumab.	Research Article cross-sectional survey.	The prices of a basket of 8 cancer drugs those with known prices in all 7 countries, was converted to US$ using both foreign exchange rates and purchasing power parity. They assessed international differences in wealth by collecting values for GDP per capita in addition to average salaries. They compared patterns of affordability of cancer drugs by dividing the drug prices by the markers of wealth GDP per capita and average salaries.	Affordability	Cancer drugs are the least affordable in India by a large margin. Despite lower prices than in the USA, cancer drugs are less affordable in MICs than in HICs. Differential pricing may be an acceptable policy to ensure global affordability and access to highly active anti-cancer therapies.
(8)	Data from 63 countries	Research Article. Cross-Sectional Survey	Online survey to evaluate (i) the availability of national formulary of licensed antineoplastic medicines across the globe, (ii) patient out-of-pocket costs for the medications, (iii) the actual availability of the medication for a patient with a valid prescription, (iv) information relating to possible factors adversely impacting the availability of antineoplastic agents and (v) the impact of the country's level of economic development on these parameters.	Availability, Access/Accessibility.	LMICs have significant lack of availability and with much less availability of new, more expensive targeted agents compared with HICs. In low-middle-income countries 32.0% of EML medicines are available only at full cost and 5.2% are not available at all, and for low-income countries, the corresponding figures are 57.7% and 8.3%. There is wide global variation in formulary availability, out-of-pocket expenditures and actual availability for most licensed anticancer medicines. Even amongst medications on the WHO EML, the discrepancies relate to high out-of-pocket costs. Overall in low-middle-income and in low-income countries reports of poor accessibility are greater. The main barriers to accessibility were either a lack of or unreliable supplier, or budgetary restraint.
(16)	23 drugs belonging to 6 different categories available in 52 different formulations were analyzed.	Research Article. Observational Study Research conducted in a tertiary care teaching hospital in south India.	The cost of anti-cancer medicine manufactured by different companies, in the same dose and dosage form, was obtained from latest issue of “Current Index of Medical Specialties” (CIMS). The difference between the maximum and minimum prices of various brands of the same drug was analyzed and percentage variation in the prices was calculated.	Pricing, Price Variations.	The average percentage variations of different brands of the same anti-cancer drug in same dose and dosage form manufactured in India is very wide. The maximum price variability was found to be highest with hormonal cancer drugs (714.24%) and lowest with targeted anti-cancer drugs (5.56%.).
(17)	Drug purchase prices for 19 national and international buyers (representing 29 total countries) were obtained from MSH.	Research article. Longitudinal analysis.	Comparative analyses were made on the median purchase prices paid (buyer price) for essential cancer medications listed on the WHO EML in the MSH (procurement dataset). Price variations was analyzed over time/date procurement was made, geography, cancer medication type, Price differentials in relation to the Disease burden of the country, GDP other therapeutic medications, generic vs. branded, dosage types were compared.	Pricing	Significant differences in prices paid across countries, regions, individual medications, and medication categories. Specifically, countries in the Africa region appeared to pay more for a package of essential cancer medication than countries in the Latin America region.
(18)	Data on 33 anti-neoplastic essential medicines collected from seven hospitals (4 public and 3 private) and 32 private-sector retail pharmacies.	Cross Sectional Study. Research Survey based on the WHO/ HAI Methodology.	Data were collected on availability and price of 33 anti-neoplastic essential medicines. Seven hospitals (four public and three private) and 32 private-sector retail pharmacies were surveyed.	Availability, Affordability, Price.	Most anti-neoplastic essential medicines were available but didn't meet the WHO target of 80%. Medicine prices were relatively low in New Delhi compared with IRPs. However, the cost of chemotherapy medicines seems unaffordable in the local context. Mean availability of anti-neoplastic EMs was 38% in private-sector retail pharmacies, 43% in public hospital pharmacies and 71% in private hospital pharmacies.
(19)	Data on cancer drug retail prices across ten countries (South-East Asia, Western Pacific and Eastern Mediterranean regions) were used in this study.	Cross-sectional survey. Research article.	Pricing data and Purchasing Power Parity (PPP)-adjusted mean unit prices for 26 anti-cancer medicine presentations (similar pharmaceutical form, strength, and pack size) were used to compare prices of anti-cancer drugs across three regions.	Pricing	There is a great variation in pricing of anticancer drugs in selected countries and within their respective regions. There was a direct relationship between income category of the countries and their mean unit price; LICs had lower mean unit prices.
(20)	Price variation was assessed for 31 anticancer medicines belonging to six broad categories in the two cancer hospitals of Nepal	Research article—cross sectional study.	The price of different brands of the same anticancer medicines available in the hospital pharmacies of two cancer hospitals was assessed. Prices of different dosage forms were calculated. The difference in the maximum and minimum price of the same drug manufactured by different pharmaceutical industries was determined, and the percentage variation in price was calculated.	Pricing, Availability.	Prices were found to vary maximally among the following medicines, each belonging to separate categories. The Government of Nepal has regulated the prices of some medicines, including anticancer medicine. However, it is not enough as prices of most anticancer medicines are still not regulated. Therefore, further strategies are needed to address the variation in the prices of anticancer medicines available in the Nepalese market. Seven medicines that were listed in the National list of essential medicines Nepal were not available in both hospital pharmacies.
(21)	Costs incurred by 50 families of children during therapy.	Research Article. Cross Sectional study	Costs incurred by 50 families of children during therapy was conducted at the Medical University Hospital in Dhaka. The patients were all treated on a modified protocol. Each family was asked to retain and submit all receipts for drugs bought from pharmacies, investigations and hospital procedures (LPs and Bone marrow sampling), transport, food and accommodation. Blood and blood products carried a standard hospital fee	Affordability	The basic cost of all treatment for each family was 3234 USD. 33% of families earned <71 USD/month, 51% between 71 and 285 USD and only 16% more than 285 USD. This means than 84% families were living on between 2 and 9 USD a day. During the period of this study treatment abandonment rates were 16%; 62% of which were reported to be due to families not being able to afford the costs.
(22)	All (*n* = 4,400) participants were ≥18 years of age.	A descriptive, cross-sectional survey	Survey was conducted in 22 cancer care hospitals (18 public hospitals and 04 private hospitals) and 44 private pharmacies in Punjab, Pakistan. to assess the availability of 40 anticancer medicines in public and private sectors, and their affordability by high, middle, and low-income class patients. The medicines were selected based on, (a) pilot study in which local needs and cancer burden was assessed, (b) literature review, and (c) the opinions of various experts.	Availability, Affordability.	The availability of both OBs and LPGs was greater at private hospitals and pharmacies as compared to public hospitals Originator brands (OBs) were more readily available (52.5%) but less affordable (53.4%); whereas, lowest price generics (LPGs) were less available (28.1%) but more affordable (67.9%). Anticancer medicines were more affordable by the HICs patients than the LICs patients.
(23)	NML for 135 (26 were LICs, 42 were lower-MICs, 44 were upper-MICs and 20 were HICs) compared with WHO's 2013 and 2015 EML.	Longitudinal Study	National medicine lists for 135 countries with per-capita gross national incomes below 25 000 United States dollars in 2015 were compared with WHO's 2013 and 2015 Model Lists of Essential Medicines. Correlations between numbers of anti-cancer medicines included in national lists and gross national income (GNI), government health expenditure and number of physicians per 1000 population were evaluated.	Availability	A regularly updated WHO Model List of essential medicines for cancer could provide guidance to countries, particularly LMICs on the most effective medicines that should be prioritized for procurement and use. Substantial numbers of anti-cancer medicines are included in national lists of LMICs.
(24)	Data collected from 18 essential and 8 ancillary antineoplastic medicines in the NEMLs or NRMLs of 135 countries with GNI per capita < US $25,000.	Research Article Cross Sectional Study	To examine the extent to which antineoplastic drugs in the SIOP, EML are included in NEMLs and NRMLs. Relationships between the numbers of medicines listed and several financial (GNI per capita, annual government health expenditure (AGHE) per capita) and workforce characteristics (the number of physicians per 1000 people) were examined.	Access	There was large variability in the antineoplastic agents identified as essential in NEMLs and NRMLs. Correlations with GNI per capita and physician density were statistically significant; not so for AGHE per capita.
(25)	Two medicines on the 2013 Thai NLEM (letrozole and imatinib) and three unlisted medicines for the same indications (trastuzumab, nilotinib and dasatinib).	Research Article. Longitudinal Study	Selected targeted oncology therapies, identified policies and programs intended to increase access to the study medicines in Thailand and assessed the utilization of targeted cancer therapies using quarterly Pharmaceutical companies and hospitals Health sales data.	Access	Government, insurance payers, and manufacturers or pharmaceutical companies implemented multi-pronged approaches to facilitate access to targeted cancer therapies for the Thai population. Utilization of the medicines and number of patients treated increased over time when the access policies were implemented.
(26)	Eight patented dugs in Mexico; bevacizumab, dasatinib, imatinib, nilotinib, rituximab, sorafenib, sunitinib, and trastuzumab.	Drug Utilization Research Method. Cross Sectional Study	Drug utilization research methods to assess the use of eight patented cancer medicines. Through the national transparency platform, data was obtained on the quantities of these medicines used in all public health facilities and social health insurance institutions in five geographic regions and recalculated those figures into defined daily dose (DDD) per 1,000 population per year.	Access	Barriers to access and use of innovative cancer medicines link to limited coverage by public insurance schemes, inclusion in the EML, availability of the medicine at the facilities, and updated clinical guidelines. Over the last 6 years, the use of eight cancer medicines has increased in Mexico, whilst the use of five has remained low due to insufficient insurance coverage. Regional differences in the use of innovative cancer medicines highlight inequalities in access to cancer care.

*GDP, gross domestic product; MIC, middle income country; HICs, high-income countries; LMIC, lower middle-income country; WHO, World Health Organization; EML, essential medicines list; CIMS, current index of medical specialties; MSH, management sciences for health; HAI, health action international; IRP, international reference price; PPP, purchasing power parity; LIC, lower -income country; LP, lowest priced; OB, originator brand; LPG, lowest priced generic; NML, national medicines list; GNI, gross national income; NEML, national essential medicines list; NRML, national reimbursable medicines list; SIOP, international society of pediatric oncology; AGHE, annual government health expenditure; NLEM, National List of Essential Medicines*.

The quality assessment shows that most of the criteria were not applicable because of the nature of the studies included in this review. Items such as ascertainment of exposure, selection of outcome, assessment of outcome were present in almost all studies whereas comparability was found to be not applicable.

The number of records included and removed at each stage was recorded in a PRISMA flow diagram (7).

### Pricing

Five studies (16–20) published on the pricing of both adult and pediatric anticancer medicines showed wide variations in prices across different countries (17–20) and regions (17, 19). There were price variations in the individual and medicine categories (17, 20) and between brands (18, 20), for example, there was the highest variation with hormonal cancer drugs (714.24%) and lowest variation with targeted anti-cancer medicines (5.56%) (16). Prices for acquiring infectious disease and cardiovascular disease medication are much lower than the median price of anticancer medicines (17). The price variation in public vs. private facilities (18, 20) was also evident.

The countries in the Africa region pay more for a package of essential cancer medicines than countries in the Latin America region (17). The median price paid for a package of cancer medicines was $12.63, with the lowest price of $0.03 and the highest price of $5250 (17). Another study estimated a cost of US$442 and US$278 to treat a 30 kg child for standard-risk leukemia and Hodgkin's lymphoma, respectively (18). Five anti-neoplastic Originator Brands (OB) were 1.2–1.4 times more expensive than their most-sold and Lowest Priced Generic (LPG) counterparts. Patients buy medicines in the private sector at 1.3 times and 2.0 times the government price and the consumer prices, respectively (18).

The Median Price Ratio (MPR) was the comparison of the local median unit price of the medicine with the median unit price in the Management Sciences for Health (MSH) 2003 International Reference Price (IRP) Indicator Guide (27). It was noted that MPR for pediatric anti-neoplastic medicines of the most sold generic, LPG and OB was 0.74, 0.71, and 1.00, which is <4 implying anti-cancer medicines in India are less expensive compared with international standards (18), as an MPR of 1 means that the medicine's price is exactly equal to the (IRP) (9).

### Affordability

Four studies (4, 18, 21, 22) showed anticancer medicines are less affordable in LMICs based on the individual patient's income approach (i.e., patients level of income/average salary) (18, 21, 22), than in High-Income Countries (HICs) based on the country's economic factor of Gross Domestic Product (GDP) per capita (i.e., a metric that breaks down a country's economic output per person) (4). Using international markers of wealth, such as the monthly GDP per Capita at Purchasing Power Parity (PPP), provided by the International Monetary Fund, the study (4) showed that prices in India, China, and South Africa were less affordable than in all HICs, including the United States (US) where prices were considerably higher.

A recent study (22) showed that high-income level patients could afford anticancer medicines better than the low-income level patients and LPGs (67.9%) were more affordable than the OBs (53.4%). Affordability studies of pediatric anti-cancer medicines (18, 21), showed that the number of days, a daily wage worker would have to work to afford cancer treatment will depend on the treatment protocol and indication. For patients with standard-risk B-cell precursor acute lymphoblastic leukemia, to buy medicines in the private retail sector, a daily wage worker earning a minimum wage of Indian Rupees (INR) 318 would have to work for 88 days (most-sold price) and 100 days (maximum retail price) and for a child with early-stage Hodgkin's lymphoma, the medicine cost would be 55 days' wages (most-sold price) and 67 days' wages (maximum retail price), respectively (18). When calculated in accordance with per-capita income, the cost of chemotherapy is 23 and 14% of per- capita income for acute lymphoblastic leukemia and early-stage Hodgkin's lymphoma, respectively (18). It was revealed that anti-cancer treatments were not affordable for most families leading to treatment abandonment (21).

### Availability

Five studies reported (8, 18, 20, 22, 23) on the availability of anticancer medicines. Some studies (18, 22) showed more availability of anti-cancer medicines in private hospitals (71%) than the public hospitals (43%), and OB (52.5%) having high availability, LPGs (28.1%) having low availability, and new anti-cancer medicines less readily available in both sectors (18, 22).

A study (8) showed substantial differences in the formulary availability and actual availability for many anti-cancer medicines. In low-middle-income countries, 32.0% of EML medicines are available only at full cost and 5.2% are not available at all, and for low-income countries (LIC), the corresponding figures are even worse at 57.7 and 8.3% (8). The medicines, on the WHO EML, are available only at full cost as an out-of-pocket expense and many of them are not available at all due to unreliable supply. There is a significant lack of availability, with much less availability of new, more expensive targeted agents (8). Lack of supplier or commercial motivation, budgetary restraint as well as unreliable supply as shown in Bangladesh, Ghana, India, Kenya, Myanmar, Pakistan, Afghanistan, and Burkina Faso was increasingly dominant (8, 18).

Other studies (20, 23) showed that substantial numbers of anti-cancer medicines are included in the National Essential Medicines List (NEML) of LMICs. The median number of anti-cancer medicines on the Model Lists that appeared on the NEML of the thirty-seven study countries in the African Region was relatively low (23). For example, of the 25 anti-cancer medicines on the 2013 Model List and the 16 added *via* the 2015 revision of the Model List, 1–23 (median: 13) and 0–14 (median: 1) appeared in national lists, respectively (23). There was considerable variability in the numbers of medicines listed within income groupings, a consistent trend was observed toward more medicines being included, as GNI per capita increased, with the median number of medicines lowest in the Africa region. What appeared on NEML differed considerably across the WB income groups, were significantly correlated with GNI per-capita, Annual Government Health Expenditure (AGHE), and the number of physicians per 1,000 population (23).

### Access

Studies on access showed large variability within income groups of what was identified as essential in NEMLs and National Reimbursable Medicine List (NRMLs) (24, 26). A study (24) explored access in 135 LMICs, to the 18 essential and 8 ancillary antineoplastic medicines proposed by the International Society of Pediatric Oncology (SIOP) to be essential in the supportive care of children with cancer. The study results focused attention on deficits in NEML and NRMLs as a step to improving access to effective antineoplastic medicines for cancers in children in LMICs.

Another study (8) observed that in India, Bangladesh, Ghana, Kenya, Myanmar, Pakistan, Afghanistan, and Burkina Faso, there was poor accessibility with patients incurring out-of-pocket cost even for generic anticancer medicines that are on the WHO EML. The dominant reported barriers to accessibility were either a lack of or unreliable supplier, or budgetary restraint. The cost and affordability of anticancer treatments with recent market approval is the major factor contributing to inequity of access to anticancer medications (8).

In studies (24, 26), there are certainly highlighted inequalities in access to cancer care. Barriers to access and use of innovative cancer medicines are linked to limited coverage by public insurance schemes, non-inclusion in the EML, non-availability of the medicine at the facilities, not updated clinical guidelines, and variability within income groups in NEMLs and NRMLs.

Another study (25) described the policy and program approaches by different health system stakeholders to facilitate access to targeted cancer therapies, which resulted in significant numbers of patients being treated with cancer medicines. Various pharmaceutical companies formed partnerships and implemented access initiatives on expanded Patient Assistance Programs (PAPs) and lowered pricing, which generally provided some form of discount or donation directly to patients enrolled in the program. The government also ensured that there were different coverage requirements and social security schemes for payers, issued compulsory licenses (CL), special marketing arrangements, and the payers negotiated prices with manufacturers and engaged in pooled procurement (25).

## Discussion

This systematic review describes a summary of the current 5 years landscape of published studies on pricing, affordability, availability, and access to anti-cancer medicines in LMICs. The breadth and depth of our review provides important understanding and appraisal of the topics as follows:

The wide price variations from the published studies (16–20) may be as a result of patent protection, monopolistic markets for new entities, regulatory issues, tax and tariffs, geographic location, income status, and lack of internal price regulation measures. Geographic location may act as a potential mediator in pricing variation, given that different prices for the same medicine are being paid by different health systems (17). Differences in guidelines of medicine regulating authorities of various countries and their pricing policies account for the varying prices of medicines among different countries (16). The existence of generics on the market might have affected originator prices in some countries, whereas in other countries originator prices remained at a high level (18). Governments should launch initiatives to promote generic prescribing by physicians, improve price transparency and empower patients to shop around for cheaper medicine prices (18).

In some developed countries, price regulation measures such as External Reference Pricing (ERP) or International Reference Pricing (IRP) have been widely used by policymakers to derive a benchmark to restrain medicine costs (28). A list of 2015 anti-cancer medicine prices by the Management Sciences for Health (MSH) based on the WHO's 21st edition of the EML (27), is the only procurement tool available to the pricing authorities in LMICs, however, more support is needed such as an updated WHO EML section on anti-cancer medicines along with cross-country pricing information and procurement guidance (19).

Greater transparency of price information among countries may assist with in-country negotiations between purchasers and suppliers. Information on the availability of cheaper medicines in neighboring countries has the potential to encourage policy and managerial decisions at national levels to reduce prices (27).

A definition of affordability is measured by the number of days' wages the lowest-paid unskilled government worker needs to spend to procure a course of treatment with medicine (9). Another definition of affordability is the comparison of medicine prices by International markers of wealth such as GDP per capita (4). Unaffordability could also refer to the percentage of the population that is already below or would fall below the poverty line when having to procure the medicine (11). There are large differences in levels of affordability around the world, with anti-cancer medicines being the least affordable in India. These differences were driven by lower levels of wealth in Middle-Income Countries (MICs). Thus, a differential pricing policy may be used to ensure global affordability (4). Precise affordability is challenging to compare between countries as there is variability as to whether medicines are publicly reimbursed, or the cost falls on the individual (4).

Affordability remains questionable as chemotherapy is required over a lengthy period incurring high total medicine costs (18, 21). Pediatric cancer therapy-related costs are dependent on the age and size of the patient which determines medicine dosage, supportive care needs, the cost of episodes of infection, and food, lodging, and travel costs. Most families with a monthly income of 70–285 USD cannot afford the high cost of treatment leading to treatment abandonment (21). The cost of generic medicines on the WHO EML (29, 30) is often not affordable in most LMICs (31). The poor affordability highlights the need to formulate policies to ensure equitable affordability, streamline public and private sector procurement and supply systems to reduce the cost to families in LMICs.

The negotiating power of small and lower-income countries is limited, consequently, affordability tends to be negatively correlated with market size and per capita GDP (32). High inflation, low per capita income, and the increasing cost of living are among the several hurdles that hinder people from affording anti-cancer medication. Differential pricing, low premium insurance schemes, medicine discounts, patient-access schemes, tax benefits, concerted public-private initiatives, patent changes, national health plans (18, 21), and emulation of salient models in governance are required for long term sustainability. The relationship between price and healthcare outcomes should be enhanced through arrangements that reward innovation while ensuring the sustainability of an affordable healthcare system (21, 33, 34).

While not a direct measure of availability, listing pediatric anti-cancer medicines on the NEMLs and NRMLs is an important step guiding procurement and the acquisition of essential anti-cancer medicines for the public sector (24). The disparities in the formulary availability and actual availability of essential anti-cancer medicines (8, 23, 24), may be due to the cost of expensive new anticancer agents (16), however some classical, low-cost, anticancer medicines, for example, tamoxifen and cisplatin, were not always routinely available largely due to governance issues, manufacturing, and distribution issues (18).

Countries with lower levels of economic development, particularly LMICs including Africa had low numbers of anti cancer medicines listed on their NEML (23). While not a direct measure of availability, the listing is an important step, guiding procurement for the public sector and thus the availability of the anti-cancer medicines (24). Efforts should be made to maintain an up-to-date list of NEMLs as an important tool to prioritize medicines and ensure their availability (23, 24).

Since chemotherapy is administered in hospitals, hospital pharmacies should ideally stock all pediatric anti-cancer medicines listed on the EMLc. However, these public hospital pharmacies had low mean availability (<80%) (18, 20) possibly due to poorly managed supply chain systems, inaccurate medicine demand forecasting, or an underfunded public health sector (35). The low availability of essential medicines in public hospitals highlights the need to streamline medicine procurement, distribution, and supply systems. Poor demand for anti-cancer medicines and high storage costs (such as refrigeration) associated with stocking these medicines might be the reason for low availability in the private-sector retail pharmacies (18).

Four studies (8, 24–26) demonstrated that barriers to access and use of innovative cancer medicines are linked to the limited coverage of public insurance schemes (26), non-inclusion in the EML (24), non-availability of the medicine at the facilities, and updated clinical guidelines (24–26). The innovation field for anti-cancer medicines is growing (36). Yet, most of the time, the high prices tagged to these innovations are not affordable for patients and health systems, thus limiting access to new cancer medicines (8). The release of affordable new and better medicines requires constant updates of treatment protocols, formularies, SIOP EML, NEMLs, and NRMLs by the anti-cancer medicines review committee as a step to improve access in LMICs (18, 24, 25).

A study showed that over 80% of the population experience barriers to accessing innovative medicines (26), that could provide them with better outcomes of their treatment against cancer. The access barriers include geographic location, inequality across insurance schemes, health care coverage (by medicine and cancer medication types), regional variations, and institutions (with the private providing more per insured population than the MoH). This could be due to differences in the burden of disease, budget and resource allocation, purchasing power, differences in capacity within the health care system, and disease priorities (17, 26, 37). The low AGHE per capita in many countries for example, Myanmar suggests that public sector procurement is likely to be problematic (24). These factors should be taken into consideration when countries assess formulary decisions, negotiate medicine procurement terms, and when formulating health and cancer policies (17).

Multi-pronged policy and program approaches by multiple stakeholders (government, payers, and pharmaceutical companies) such as efficient resource allocation (26), decentralization of health care, patient assistance programs, special marketing arrangements, and issuance of compulsory licenses for procurement will facilitate equitable access and use of effective and affordable cancer treatments (25, 26). Improving access with innovative treatments of which the effectiveness, safety, and cost-effectiveness have been established, will provide a better quality of cancer care, better health outcomes, and fewer deaths due to cancer (38).

Further observations and critique on included studies showed that there was no in-depth analysis of each country's respective health care system to understand the price differences and what they mean in terms of access to cancer medications, government/public spending, and patient adherence (19). There was the inaccessibility of confidential discounted prices, and thus savings for payers were not explored (19). The use of retail prices, which include add-ons such as taxes and distribution fees had limited data on the add-ons, and thus it was not estimated, to understand the sources of add-ons to identify potential targets for price reduction (19). The price variations among formulations containing a combination of medicines and independent variables were not analyzed and there was a lack of comparison with the prices of many anti-cancer medicines manufactured by different companies (17, 18).

There are some limitations with the studies conducted on affordability, namely other economic factors that can influence the affordability of anti-cancer medicines (18, 21, 22) were not assessed. Using monthly costs (4, 22) may be less appropriate than using total treatment costs (18, 21). The non-randomized selection of countries in the analysis limits the ability to extrapolate these data to the whole world (4, 18, 21, 22).

There are some limitations with the studies conducted on availability: namely, it could not capture the pattern of medicine availability over time as availability was measured at one time on the day of data collection from the health facilities. Availability may be better understood through a longitudinal study instead of a cross-sectional study (18, 22). For each study country, there was no investigation on the actual availability of the listed medicines on national documents or lists applicable to specialist cancer facilities (23).

A couple of limitations were observed with the studies on access. In this study (24) the EMLs or NRMLs were not available for some countries, some available documents were outdated, and there may have been more recent versions of the documents not included in the sources used. The gaps in EMLs or NRMLs may hinder access to effective antineoplastic medicines in countries. This study (26) focused on a selected number of innovative cancer medicines and does not account for the whole treatment scheme. Further research should focus on complete treatment schemes to inform stakeholders and policymakers on the current situation and identify potential access barriers to be addressed. With this study (25), the differences in estimated numbers of patients treated based on differences in product volumes sold could have occurred because of changes in therapeutic regimens over time, general market growth, or the complexities of supply systems and stock management. Continued research is needed to assess the challenges in accessing these medicines at the household and system levels.

## Limitations with the Literature Review

Investigations were limited to the English language literature, thus publications in other languages were not included. Also, relevant conference abstracts were excluded from the systematic search. The small number of articles (13) included in the qualitative synthesis limited our ability to conclude wide and comprehensive conclusions. The use of publications in the last 5 years was to emphasize the most relevant data available in this field that reflect the current situation in countries and to avoid data that is outdated, less relevant and not reliable, even though this narrowed the relevant population. Lastly, the review study selection included only articles published in peer-reviewed journals, gray literature was excluded. This was to ensure an academic level of accuracy through the peer-review process. Despite this limitation, the review provides important insights into the pricing, availability, access and affordability of cancer medicines in LMICs.

## Conclusions

This systematic review summarizes recent original research on the topic of cancer pricing, availability, affordability, and access in LMICs. It showed that in LMICs, there are wide variations in cancer prices with less affordability by patients with low-income levels. Barriers to access and use of cancer medicines are linked to the high cost of cancer medicines, limited coverage by public insurance schemes, non-inclusion in the EML, and limited or non-availability of the medicine at the facilities.

This review illustrates the dearth of information regarding how cancer medicines are priced in Africa and other developing countries. It showed that the studies conducted have different themes from one another, with a few having combined themes and outcomes. None addressed all the four parameters of pricing, affordability, availability, and access. With the emerging themes and limitations noted, further research studies holistically addressing issues on pricing, availability, affordability, and access to anti-cancer medicines in LMICs especially in Africa should be undertaken.

## Data Availability Statement

Publicly available datasets were analyzed in this study. This data can be found at: Medline/CINAHL EBSCO, PUBMED, Web of Science, Google Scholar, Springer link, and Scopus databases.

## Author Contributions

PO and Z-U-DB: conceptualization and formal analysis. PO, Z-U-DB, and SH: methodology. PO: writing original draft preparation. RA, SH, and Z-U-DB: writing review and editing. Z-U-DB: supervision. All authors have read, approved, and agreed to the published version of the manuscript.

## Conflict of Interest

The authors declare that the research was conducted in the absence of any commercial or financial relationships that could be construed as a potential conflict of interest.

## Abbreviations

LMIC: lower middle-income country
HER2+: human epidermal growth factor Receptor 2 positive
WHO: World Health Organization
EML: essential medicines list
EMLc: essential medicines list for children
PRISMA: preferred reporting items for systematic reviews and meta-analyses
GNI: gross national income
WB: world bank
NOS: newcastle-ottawa scale
OB: originator brand
LPG: lowest priced generic
MPR: median price ratio
MSH: management sciences for health
IRP: international reference price/pricing
HICs: high-income countries
GDP: gross domestic product
US: United States
PPP: purchasing power parity
LIC: low income countries
NEML: national essential medicines list
AGHE: annual government health expenditure
NRML: national reimbursable medicines list
SIOP: international society of pediatric oncology
ERP: external reference price
MIC: middle income country
INR: indian rupees
CL: compulsory license.
